# Reducing bias in experimental ecology through directed acyclic graphs

**DOI:** 10.1002/ece3.9947

**Published:** 2023-03-28

**Authors:** Suchinta Arif, Melanie Duc Bo Massey

**Affiliations:** ^1^ Department of Biology Dalhousie University 1355 Oxford Street Halifax Nova Scotia B3H 4R2 Canada

**Keywords:** causal inference, collider bias, confounding bias, directed acyclic graphs (DAGs), external validity, overcontrol bias, randomized control trials (RCTs)

## Abstract

Ecologists often rely on randomized control trials (RCTs) to quantify causal relationships in nature. Many of our foundational insights of ecological phenomena can be traced back to well‐designed experiments, and RCTs continue to provide valuable insights today. Although RCTs are often regarded as the “gold standard” for causal inference, it is important to recognize that they too rely on a set of causal assumptions that must be justified and met by the researcher to draw valid causal conclusions. We use key ecological examples to show how biases such as confounding, overcontrol, and collider bias can occur in experimental setups. In tandem, we highlight how such biases can be removed through the application of the structural causal model (SCM) framework. The SCM framework visualizes the causal structure of a system or process under study using directed acyclic graphs (DAGs) and subsequently applies a set of graphical rules to remove bias from both observational and experimental data. We show how DAGs can be applied across ecological experimental studies to ensure proper study design and statistical analysis, leading to more accurate causal estimates drawn from experimental data. Although causal conclusions drawn from RCTs are often taken at face value, ecologists are increasingly becoming aware that experimental approaches must be carefully designed and analyzed to avoid potential biases. By applying DAGs as a visual and conceptual tool, experimental ecologists can increasingly meet the causal assumptions required for valid causal inference.

## Graphical abstract

Randomized control experiments (RCTs) are also vulnerable to common biases found throughout observational studies, including confounding, overcontrol, and collider bias. Using directed acyclic graphs (DAGs) can help researchers avoid bias and lead to a better causal understanding of natural phenomena through RCTs.

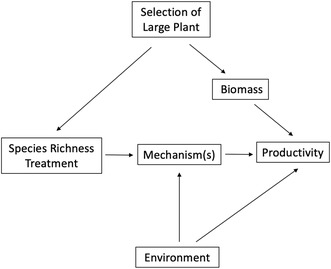

## 1 INTRODUCTION

Experiments are a fundamental tool ecologists use to quantify causal relationships, in particular, by using randomized control trials (RCTs) that often regarded as the “gold standard” for causal inference (e.g., Hariton & Locascio, 2018). Under RCTs, researchers randomly assign units or individuals into treatment and control groups to eliminate potential confounding between treatment assignment and outcome, thereby increasing the internal validity of experiments (Holland, 1986; Rubin, 1974). Many of our foundational insights in biology were discovered through experiments. For instance, during early exploration of the scientific method, Francesco Redi (1626–1698) famously conducted his “fly experiments” to test the theory of spontaneous generation (Gottdenker, 1979). Redi designed an experimental setup in which there were eight identical flasks containing meat; he tightly sealed four of these flasks and left four uncovered, yielding “treatment” and “control” groups (Gottdenker, 1979). In contrast to previously held beliefs that maggots were created within dead flesh itself, Redi's experiment revealed that only that meat which was exposed to incoming flies would eventually produce maggots, drawing the causal conclusion that, for maggots to form, “live animals must… deposit their seeds” (Gottdenker, 1979). In addition to the fundamental observation *omne vivum ex vivo* (“all life comes from life”), such experimentation would ultimately cascade into more complex tests of the causal relationships in the natural world.

Although RCTs have been invaluable in understanding numerous causal relationships in ecology, they are nonetheless susceptible to biases that can lead to erroneous causal conclusions. For example, Kimmel et al. (2021) discuss core causal assumptions required for valid causal inference in experimental biology. This includes excludability, which is the assumption that the process by which treatments are assigned has no effect on the outcome. Other studies have noted that RCTs can suffer from lack of generalizability, for example, because ecological treatments may not accurately represent actual ecological phenomena (e.g., Korell et al., 2019). Further, statistical approaches that have recently received criticism across observational ecological studies are also prevalent across experimental ecological studies. For example, many experimental studies employ predictive model selection techniques such as Akaike's information criterion (AIC; Akaike, 1973) to select the best model for analysis (e.g., Cameron et al., 2013; Hunyadi et al., 2020; Sato et al., 2011); others place all predictor variables of interest as well as potential confounders into one statistical model for analysis (McElreath, 2020). Such approaches have been shown to be unreliable for drawing causal conclusions (Arif & MacNeil, 2022a; McElreath, 2020). Although causal conclusions drawn from RCTs are often not questioned, biases may still arise, either through study design and/or statistical analysis. However, there is currently no unified framework that is being employed to ensure accurate causal conclusions are drawn across RCTs in ecology.

The structural causal model (SCM; Pearl, 2009) is a causal inference framework that has recently been highlighted in the ecological literature as a tool for determining causal relationships from observational data (Arif et al., 2021; Arif & MacNeil, 2022a, 2022b; Laubach et al., 2021; Schoolmaster et al., 2020). The SCM framework uses directed acyclic graphs (DAGs) to visualize hypothesized causal relationships between variables of interest, identify potential biases, and guide appropriate study design and statistical analysis required for causal inference. What has received significantly less attention is that this framework can also be used to reduce bias across RCTs (e.g., Schoolmaster et al., 2020). Here, we overview how DAGs can reduce common biases across RCTs and advocate for their widespread uptake across experimental studies.

## 2 THE SCM FRAMEWORK

The SCM framework uses DAGs to represent the causal structure of a system or process under study. DAGs consist of variables (nodes) that are connected to each other via directed arrows, pointing from cause to effect. These directed arrows communicate a causal relationship between two variables but make no assumptions about the functional form or effect size (Glymour & Greenland, 2008). DAGs must also include both measured and unmeasured variables required to depict the complete causal structure of a system or process (see Cronin & Schoolmaster Jr, 2018; Schoolmaster et al., 2020 for complete examples of ecological DAGs).

**FIGURE 1:**
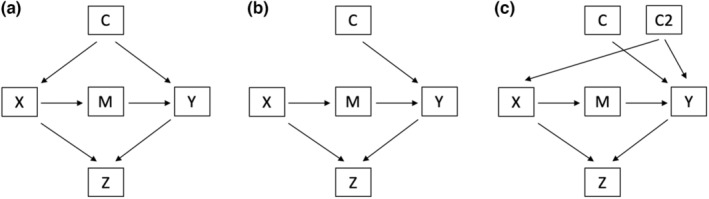
Three directed acyclic graphs representing (a) the causal structure in a natural setting, with a confounder (c) affecting both the variable of causal interest X and the outcome Y (b) the causal structure under a perfectly executed randomized control trial (RCT), which breaks the association between C and X and (c) the causal structure under a RCT that introduces additional confounding from variable C2.

As an example, Figure 1a shows a DAG representing a natural system whereby X affects Y through mechanism M, C affects both X and Y, and both X and Y affect Z. Here, to determine the effect of X on Y, we can apply a graphical rule known as the *backdoor criterion* to determine which variables need to be controlled for to answer our causal question (Pearl, 2009). Specifically, the backdoor criterion instructs us to block all backdoor paths between X and Y (i.e., our predictor and response variable of interest). Backdoor paths are sequences of nodes and arrows between X and Y with an arrow pointing into X; if left open, they can induce spurious (noncausal) associations between X and Y, biasing estimates. To block a backdoor path, we can either (1) control for an intermediate arrow‐emitting variable or (2) not control for a variable with two incoming arrows (i.e., a collider variable, such as Z) in the pathway. To determine the effect of X on Y, there is one backdoor path that needs to be blocked: X ← C → Y (Figure 1a). To block this pathway, we can control for the arrow‐emitting variable C. There are several ways to control for a variable, including experimental control, as well as statistical techniques including covariate adjustment, stratification, and restriction (Williams et al., 2018).

The backdoor criterion removes noncausal associations that often plague observational studies, including confounding, collider, and overcontrol bias. *Confounding bias* occurs when a variable that affects both the predictor and response variable is not controlled for. Given our DAG in Figure 1a, to determine the effect of X on Y, we must control for C to remove confounding bias. Here, not controlling for C would leave the backdoor path (X ← C → Y) open, leading to a noncausal association between X and Y. *Collider bias* occurs when both predictor and response affect a third common variable (or its descendant), and that variable (known as a collider variable) is controlled for. To determine the effect of X on Y, we must avoid controlling for Z. Here, controlling for Z opens a noncausal pathway (X → Z ← Y), leading to noncausal associations between X and Y (Figure 1a). *Overcontrol bias* occurs when an intermediate variable along a causal pathway between predictor and response is controlled for, blocking the indirect causal association between treatment and response. To determine the effect of X on Y, we must not control for M (Figure 1a). Here, controlling for M closes a causal pathway (X → M → Y), removing this causal association between X and Y.

A perfectly designed RCT should remove all backdoor paths between treatment and outcome. Figure 1b represents our previous DAG under a perfect RCT where treatment X is controlled and randomized. The arrows pointing into X are removed under the assumption that only the experiment determines the value of X. Under this scenario, there are no backdoor paths that need to be blocked (because C no longer affects X), and the effect of X on Y can be estimated without bias. However, ecological experiments can often diverge from perfectly executed RCTs (Kimmel et al., 2021; Schoolmaster et al., 2020; Williams et al., 2018) and backdoor paths may be open, for example, due to additional confounding variables that arise from an imperfect treatment assignment process. For example, in Figure 1c, the treatment assignment process led to an additional confounding variable, C2, that affected both treatment assignment X and outcome Y. Bias can also arise from improper statistical analysis of experimental data. For example, controlling for M in Figure 1b,c will lead to overcontrol bias, whereas controlling for Z in Figure 1b,c will lead to collider bias. By visualizing ecological experimental setups through DAGs, researchers can ensure that common biases are accounted for, allowing for more accurate causal conclusions to be drawn from experiments.

Below we showcase how describing an experimental design via a graphical DAG can reveal confounding, overcontrol, and collider bias. In doing so, we also show how DAGs can be used to eliminate these biases in RCTs, which can be done during the experimental design process and the statistical analysis stage. We further show how the drawing of DAGs at the experimental design phase can be used to assess external validity, focusing on the extent to which RCTs can be generalizable to real‐world scenarios. The case studies and associated DAGs depicted in this paper are simplified and used for illustrative purposes. We refer readers to Arif and MacNeil (2022b) for a comprehensive overview of creating complete DAGs for ecological research.

## 3 CONFOUNDING BIAS

Ecologists are well aware that confounding bias can often plague observational correlative studies; however, with RCTs it is assumed that the randomization process will eliminate confounding. To break any confounding between treatment assignment and outcome, the excludability assumption must be met (Kimmel et al., 2021). Excludability assumes that the process by which treatments are assigned has no effect on the outcome except through its effects on variation in treatment. However, the process of treatment assignment across ecological experiments can often lead to the excludability assumption being violated, subsequently leading to confounding bias; below we present two examples:

### 3.1 Biodiversity–Ecosystem function experiments

Hundreds of experiments have been carried out to understand the causal relationship between biodiversity and ecosystem function (Loreau et al., 2001). Although the drivers of ecosystem functioning are numerous and often interconnected, BEF studies rarely communicate the causal structure of their study system, or the causal assumptions required for their experimental setup. However, both are necessary as biodiversity–ecosystem function (BEF) experiments may be prone to erroneous conclusions (Schoolmaster et al., 2020).

As a classic example, the Cedar Creek grassland experiments (Tilman et al., 1996) sought to determine the effect of plant species richness on productivity. Each experimental unit was a plot containing 1–24 species that were planted from seeds, forming the species richness treatment. The community of species within each plot was established by randomly drawing from a pool of 24 prairie species. A given species had a 1/24 chance to be drawn into a treatment with a species richness of 1, a 6/24 chance of being drawn into a treatment with a species richness of 6, and so forth. It was thus assumed that the community within each plot was fully “randomized.” Care was taken to ensure plots were otherwise similarly treated (i.e., free of previous wild vegetation, consistent and equal weeding) throughout the experiment. After two growing seasons of experimental maintenance, Tilman et al. (1996) sampled plant biomass and concluded there was a positive causal relationship between species richness and productivity.

**FIGURE 2:**
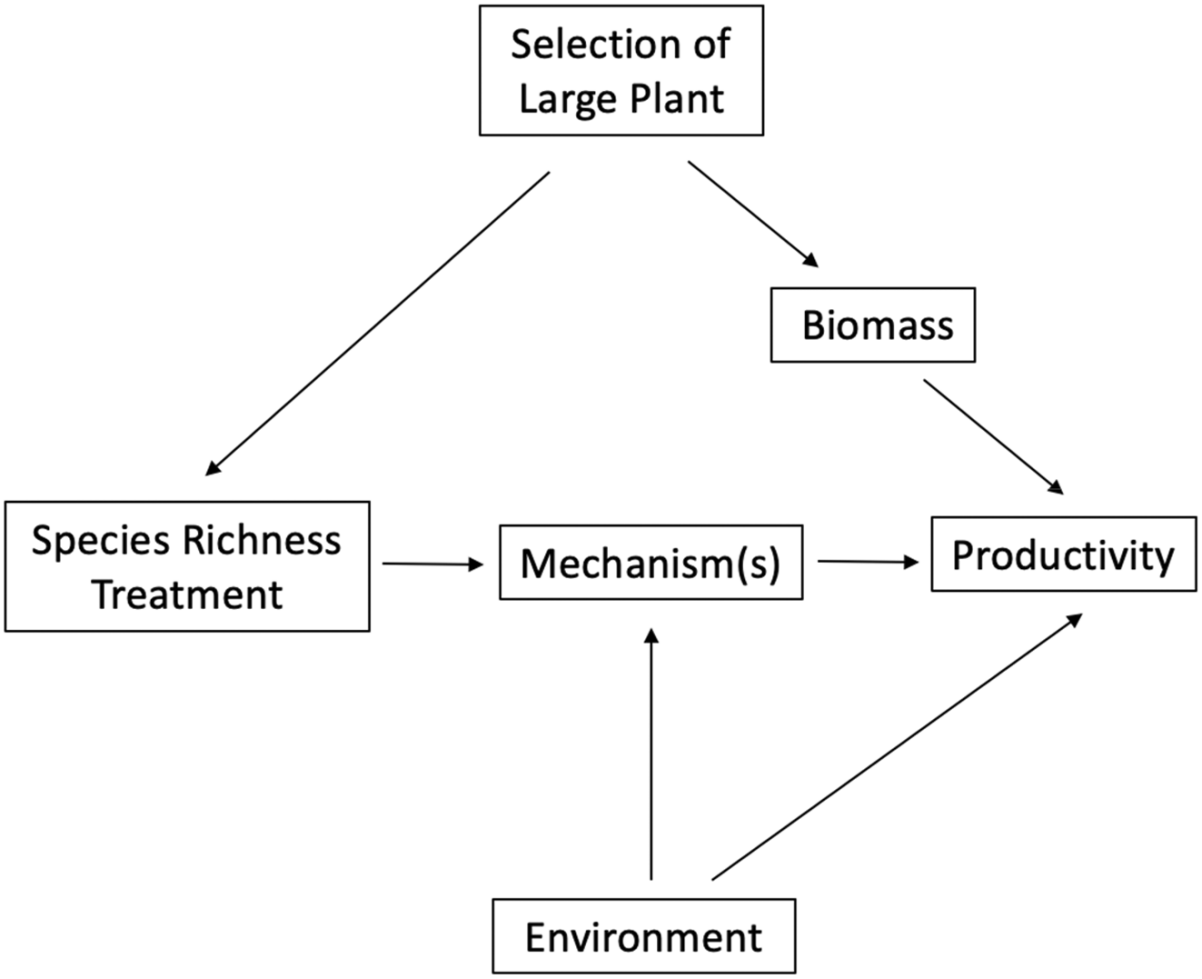
Simplified directed acyclic graph representing confounding bias in a biodiversity–ecosystem function (BEF) experiment. The directed arrow from environment to species richness, which would otherwise exist in nature is removed due to the experimental treatment assignment process. However, the treatment assignment process induced an additional confounding variable, whereby selection of large plants into a treatment differentially affected high vs. low species richness treatments as well as affected the productivity outcome.

Although this experiment was carefully designed, a subtle bias known as the “‘selection probability effect” may have confounded the results of this study (Figure 2; Huston, 1997). The selection probability effect occurs when there is an increasing chance of selecting a species with a specific trait as the number of sampling events increases. In this study, the selection probability bias was evident as size variation existed among the 24 candidate species, and large species were more likely to be drawn into high species richness treatment plots, differentially impacting treatment assignment (Figure 2). Further, since plant communities are typically dominated by individuals from large species, productivity data gathered from treatments with overrepresentation of large, dominant plants likely reflected the effects of those dominant species, rather than species richness itself (Huston, 1997). Thus, the positive correlation between species richness and productivity found in this study may be due to confounding bias resulting from large plant species affecting both treatment assignment and outcome (Figure 2). Graphically, this is represented by a backdoor path between treatment assignment and productivity (productivity ← biomass ← selection of large plant → species richness treatment) being left open, leading to confounding bias. Although other issues with this study have also been noted (Huston, 1997), this issue could be resolved by a study design that samples from plant species of similar height, removing the selection probability effect.

Experiments continue to inform our understanding of BEF correlations. At the same time, some authors have highlighted biases that may arise across BEF experiments (e.g., Huston, 1997; Kimmel et al., 2021; Mora et al., 2014; Schoolmaster et al., 2020). The future uptake of DAGs within BEF studies can transparently communicate the overall causal structure of a study system as well as identify any potential biases that may be at play.

### 3.2 Transgenerational experiments

In recent years, experimental biologists have increasingly placed emphasis on phenotypic plasticity as a means of coping with climate impacts (e.g., Seebacher et al., 2015). One form of phenotypic plasticity that is expected to contribute to organismal responses is transgenerational plasticity (TGP), whereby ancestral environments influence the phenotypic responses of subsequent generations nongenetically (Donelson et al., 2018; Salinas et al., 2013).

Transgenerational experiments are necessarily complex, given that an ancestral (F0) generation must be reared to sexual maturity under the desired conditions, reproduce, and then the responses of subsequent (F1, F2, etc.) generations must be recorded. Throughout the experiment, there is a risk of unexpected variables impacting the assignment of individuals into the F1 (or later) treatment group and response simultaneously.

**FIGURE 3:**
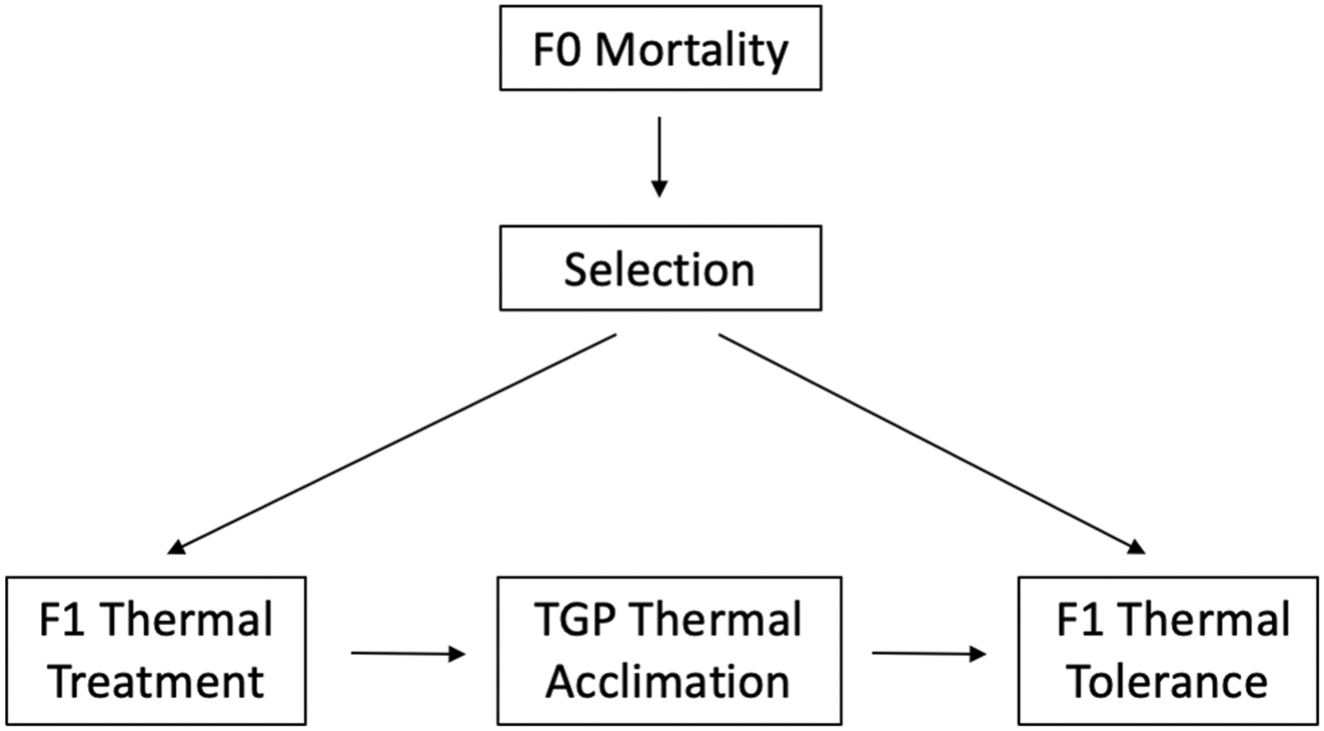
Directed acyclic graphs representing a transgenerational plasticity experiment, whereby differential mortality under the ancestral (F0) generation treatment leads to selection differentially affecting the subsequent (F1) treatments.

If selection exerts significant effects on both treatment assignment and response, an over or underestimation of the strength of plasticity effects can occur. As an example, Zizzari and Ellers (2014) investigated TGP of heat tolerance in a collembolan arthropod. They exposed F0 females to a significant heat shock and then estimated the heat tolerance of F1 offspring (Zizzari & Ellers, 2014). A notable result of this study is that the mortality rate of heat‐shocked F0 mothers was greater than double of control mothers (19% vs. 8%). Mortality of F0 fish differentially affected selection across F1 treatment group, with potentially higher selection in the heat shock treatment (Figure 3). Selection may have also affected the outcome of interest, with higher selection increasing F1 thermal tolerance (Figure 3). Graphically, the backdoor path between F1 thermal treatment and F1 thermal tolerance (F1 thermal tolerance ← selection → F1 thermal treatment) is left open, leading to confounding bias. Thus, F1 offspring whose mothers had greater genetic capacity to tolerate heat shock may have been overrepresented in the heat shock treatment, potentially leading to an overestimation of the strength of TGP.

In such cases as these, researchers at a minimum should be explicit in acknowledging whether their experimental treatments were subject to differential selection, and clearly rationalize how selection may have affected their conclusions (e.g., Donelson et al., 2018). Authors can also opt to reduce differential selection by decreasing the magnitude of treatment (e.g., reducing treatment‐induced stress). Authors should be conscientious in recording treatment‐dependent metadata (e.g., mortality) to make informed decisions about potential confounders.

## 4 COLLIDER BIAS

Collider bias occurs when both the treatment and outcome each affect a third “collider” variable (or its descendant), that when controlled for, leads to a noncausal association between treatment and outcome. A common way for collider bias to occur under RCTs is if both the treatment and outcome affect whether an individual or unit is included in the final analysis of a study. For example:

### 4.1 Survivorship bias

Data analyzed from RCTs are sometimes limited to individuals or units that have survived the full term of an experiment. As an example, Lusk and Del Pozo (2002) conducted an experiment to quantify the growth rates of plant species under low‐light and high‐light environments. Seedlings from 12 Chilean rainforest tree species were grown under both low‐ and high‐light environments, and relative growth rates (RGR) of individual plants were measured 5–6 months following the start of the experiment. Their results showed that RGR in high‐light treatment were consistently higher than low‐light treatment across all species.

**FIGURE 4:**
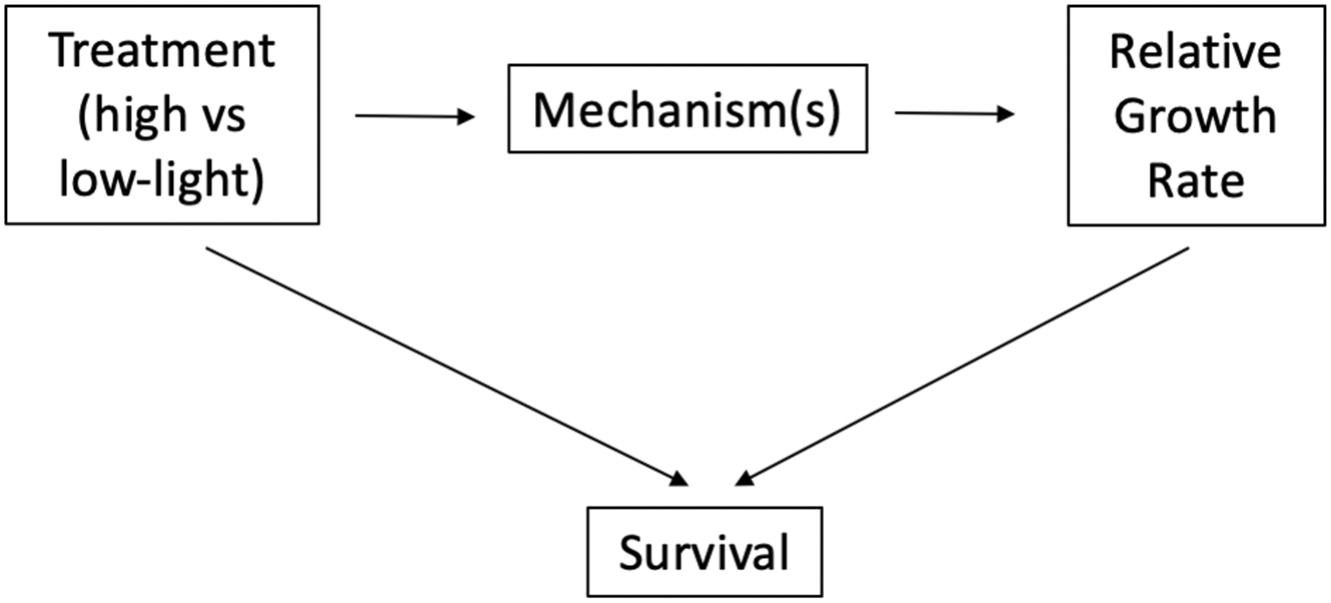
Simplified directed acyclic graph representing collider bias in a randomized control trial. Here, both the treatment assignment (high vs. low‐light conditions) and the outcome (relative growth rate) affected which plant individuals survived until the end of the experiment. Only analyzing data from plants that survived until the end of the experiment will essentially control for this collider variable. This in turn will induce a noncausal spurious correlation between treatment and outcome, leading to collider bias.

The study noted that mortality rates were significantly higher in low‐light conditions. As well, mortality risks tend to be higher for slow‐growing plants (i.e., those with lower RGR) in a population (Kobe et al., 1995). Thus, both the treatment status (low‐ vs. high‐light) and RGR outcome affected whether an individual plant survived long enough to be included in the final analysis (Figure 4). In other words, the collider variable “survival” (representing plants that survived until end of experiment) was controlled for, leading to a noncausal association between treatment and outcome (Figure 4). Here, low‐light growth rates may be overestimated, as only the “winners” from low‐light conditions were assessed.

Analyzing any subset of experimental data may lead to collider bias if the subset of data is affected by both the treatment and response. A second example is overviewed in Williams et al. (2018), where an RCT investigating the effect of promoting breastfeeding on child cognitive development led to collider bias when only participants who attended a post‐treatment follow‐up were analyzed; here, both the treatment assignment and outcome affected the likelihood of participants following up. In general, researchers should be conscientious not to control for post‐treatment variables that are influenced by both the treatment and outcome.

## 5 OVERCONTROL BIAS

Overcontrol bias occurs when an intermediate variable along a causal pathway between treatment and outcome is controlled for. Unlike confounding and collider bias, which induces noncausal associations, overcontrol bias removes indirect causal associations between treatment and outcome.

### 5.1 Intermediate variables in temperature experiments

Temperature is one of the main drivers of biological functions, influencing biotic enzyme kinetics, whole‐organism physiology, population growth and distribution, and species interactions (e.g., Wieser, 1973). Given the numerous ways in which temperature can affect an outcome of interest, it is crucial to understand when variables act as mechanisms along a causal pathway between temperature treatment and outcome, as controlling for such variables can lead to overcontrol bias.

As an example, Lienart et al. (2014) conducted an experiment examining the impact of temperature and food availability on risk behavior in fish. They collected wild juvenile *Pomacentrus chrysurus* and then randomly allocated them to one of four treatments, each representing a combination of two feeding levels and two temperature treatments. After 5 days of acclimation under experimental conditions, risk behavior was assessed.

**FIGURE 5:**
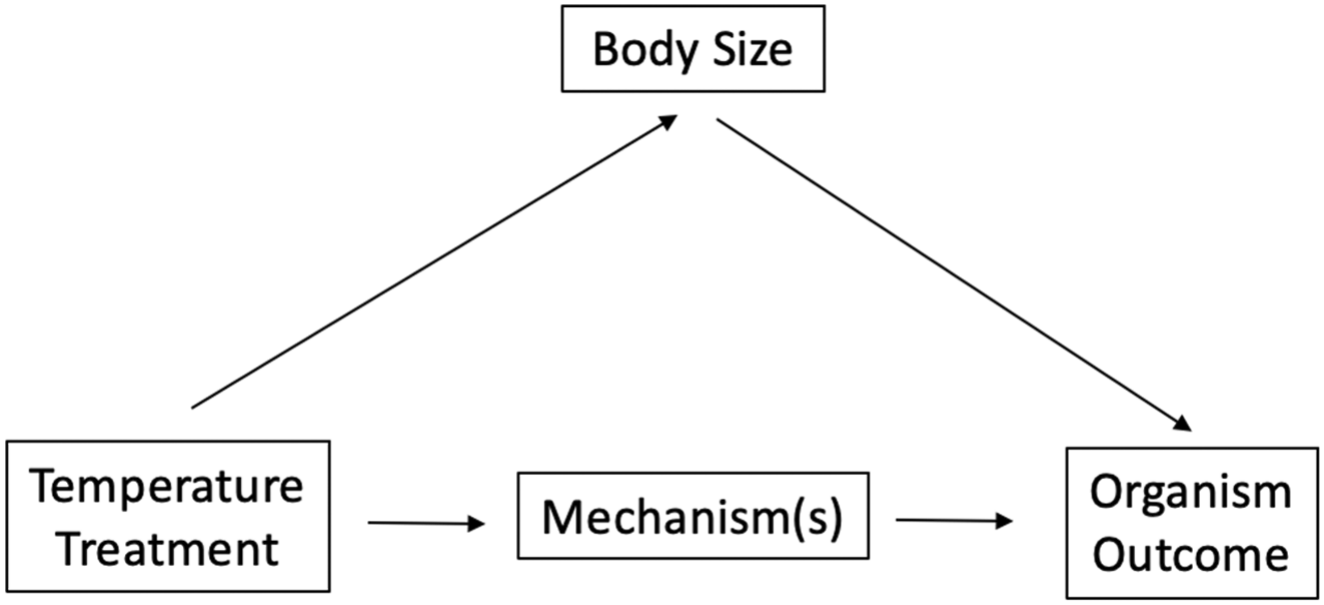
Generalized directed acyclic graph representing the effect of temperature treatments on an organism outcome (e.g., risk behavior). Here, body size acts as an intermediate variable between treatment and outcome. If body size is controlled for, it will lead to overcontrol bias, removing this indirect causal association between treatment and outcome.

To determine the effect of temperature and food on risk behavior, the authors included size as a covariate in their statistical analysis, nothing that “the manipulation of both temperature and food could have resulted in a difference in the size of the fish, which could potentially affect their antipredator response.” Their rationale implies that body size acts as an intermediate variable between treatment and response (Figure 5) and should not be controlled for to determine the total effect of treatment on outcome. Although the authors noted a negligible effect of size, this study nonetheless highlights the misconceptions that experimental ecologists may have about statistically controlling for mechanisms along causal pathways.

As a second example, Spinks et al. (2020) conducted an RCT to investigate the parental effect of warming on reproduction and offspring quality. In their analysis, they decided whether to include post‐treatment mother size (an intermediate mechanism between treatment and outcome) as a covariate based on the leave‐one‐out cross‐validation information criterion (LOOIC), a predictive model selection technique. However, model selection techniques are meant for predictive inference (i.e., what is the best model to predict Y?) and should not be conflated with causal inference (i.e., what is the effect of X on Y?). In fact, predictive model selection techniques can often lead to overcontrol as well as other forms of bias (Arif & MacNeil, 2022a).

Additionally, researchers sometimes include an intermediate variable as a covariate because they are also interested in their causal effect on the outcome. For example, the influence of body size may be of fundamental biological interest, even if the influence of temperature is the primary question in the study (e.g., Fuxjager et al., 2019). However, in such cases, a separate causal model should be built for each variable of interest, following the backdoor criterion. Given our DAG in Figure 5, to determine the effect of treatment on outcome, no additional covariates need to be controlled for, as there are no backdoor paths that need to be blocked. However, to determine the effect of body size on outcome, the backdoor path outcome ← mechanism → treatment → body size can be blocked by either controlling for “treatment” or “mechanism.” This could be achieved, for example, by statistically adjusting for either treatment or mechanism.

As a correct example, an experimental study investigating the effects of ocean warming in marine sticklebacks noted that they “did not include egg size as a covariate as egg size is an intermediate variable that may have been affected by temperature treatments in the F0 and F1 generations” (Shama & Wegner, 2014). Here, authors recognize that statistically controlling for a mechanism should be avoided if looking for the overall effect of a treatment on outcome. Statistically controlling for an intermediate variable is also valid if researchers are not interested in that particular causal pathway. For example, if we wanted to know the direct (vs. total) effect of temperature on outcome, then given our DAG in Figure 5, we would statistically control for body size to remove the causal effect of this indirect pathway (temperature treatment → body size → organism outcome).

A formal method for acknowledging and avoiding overcontrol bias can benefit experimental ecologists and lead to more informed experimental conclusions. DAGs allow researchers to visualize when variables may act as part of a causal pathway, subsequently allowing them to justify their exclusion or inclusion as a covariate (i.e., statistical control) in their analysis.

## 6 EXTERNAL VALIDITY

External validity represents the degree to which results of an experiment can be generalized to subjects and situations outside of the experimental setup (Shadish et al., 2002). Ecologists have previously highlighted the ways in which external validity can be increased across RCTs, for example by conducting field experiments that are employed under natural settings, or replicating experiments across settings, populations, and conditions to determine whether results can be generalizable. Here, we highlight how DAGs can be used to visualize how experimental conditions may systematically differ from real‐world conditions, and how this in turn can affect the causal conclusions drawn from experimental studies.

### 6.1 The obfuscating influence of static treatments

In nature, abiotic conditions such as temperature, dissolved oxygen, salinity, moisture, and light are rarely static; instead, they vary both temporally (diurnally, seasonally, and stochastically) and spatially. Despite this, experiments often compare static treatment conditions against one another, potentially resulting in data that lack ecological relevance, or are otherwise obfuscated by static condition‐imposed pathologies.

**FIGURE 6:**
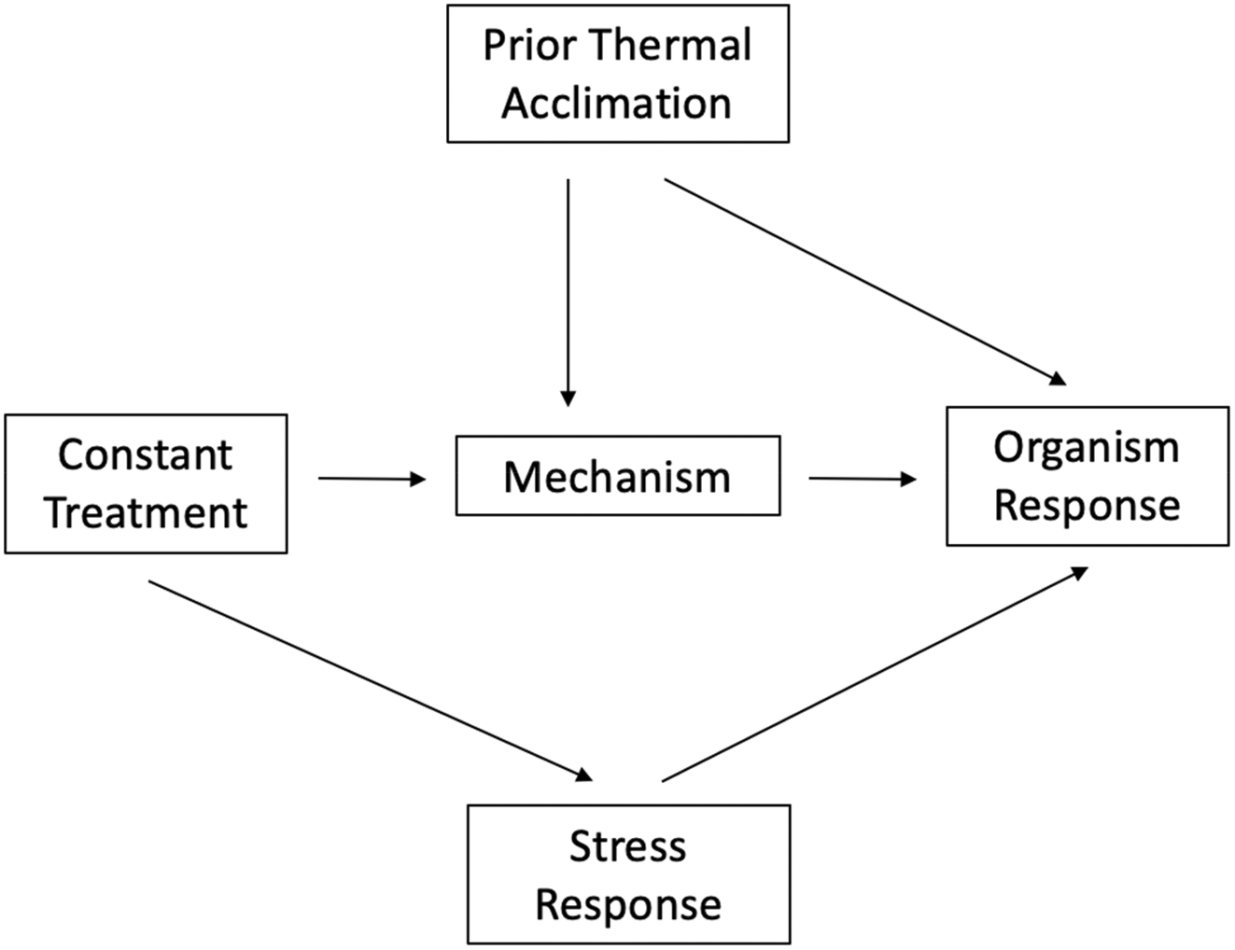
Generalized directed acyclic graph representing how, in thermal biology, both prior thermal acclimation of an organism to static conditions and stress responses induced by static treatment conditions can influence outcomes, and thus the external validity of experiments.

For example, several lines of evidence suggest that the use of constant temperatures may have serious repercussions on both individuals and even entire populations of experimental organisms. For instance, a recent study by Morgan et al. (2020) found that the laboratory‐reared zebrafish, which have been kept at constant 28°C for over 150 generations, have significantly limited capacity to plastically respond to thermal conditions when compared to lines of wild‐caught zebrafish. Moreover, this acclimation capacity was limited at all levels of biological organization, from genetic, to physiological, to behavioral plasticity (Morgan et al., 2020). These findings ultimately challenge the generalizability of conclusions garnered from lab‐reared zebrafish, a model organism used in at least 17,151 studies as of 2013 (Kinth et al., 2013). In this situation, the evolutionary history of thermal adaptation to unnatural constant temperatures may affect the mechanisms and responses to treatments (Figure 6). Here, although the treatment effect can be quantified without bias (i.e., there are no open backdoor paths between treatment and organism response), the causal conclusions drawn will not be generalizable to what would be expected under a natural setting. The generalizability of conclusions from these experiments will thus depend on the strength of the effects of prior thermal adaptation or acclimation, and researchers should think critically about how recorded responses may differ from those in the natural system they are trying to represent.

The generality of constant temperature experiments has also widely been criticized due to the possibility of treatments imparting unintended pathologies, especially under stressful conditions (Massey & Hutchings, 2020). Natural organisms have evolved to respond to changing environments and consequently are expected to perform better when a stressor is applied at a natural time scale rather than through chronic exposure (Angilletta Jr & Angilletta, 2009; Colinet et al., 2015). For example, Kingsolver and Woods (2016) demonstrated that at hot constant temperatures, organismal growth becomes limited due to pathological increases in molecular coping mechanisms (e.g., heat shock proteins), which reduces energy resource availability in the growth pathway. When a model based on constant temperature performance is subsequently applied to estimate growth under natural, fluctuating temperatures, growth is underestimated (Kingsolver & Woods, 2016; but see also Rollinson et al., 2018). Therefore, the outcomes measured in constant temperature treatments may themselves be subject to the influence of additional and unintended mechanisms such as a stress response (Figure 6) and do not reflect what is expected in nature.

Although approaches that modify constant temperature models to extend their applicability have been developed (e.g., controlling for Stress Response in Figure 6; Kingsolver & Woods, 2016; Koussoroplis et al., 2017), many authors now advocate for the use of more ecologically relevant temperature regimes in experimental biology as a means of generating realistic responses and conclusions (e.g., Massey & Hutchings, 2020; Morash et al., 2018; Taylor et al., 2021).

## 7 CONCLUSION

Directed acyclic graphs are starting to gain traction across ecological observation studies but have yet to be applied in experimental ecology. Although causal conclusions drawn from RCTs are often taken at face value, ecologists are increasingly becoming aware that for causal inference to be valid, experimental approaches must be carefully designed and analyzed to avoid potential biases (Kimmel et al., 2021). By routinely using DAGs, researchers can avoid biases including confounding, collider, and overcontrol bias across experimental studies. DAGs can also assess external validity of experiments by visualizing how mechanisms may differ between experimental setups and the natural world. By applying DAGs as a visual and conceptual tool, experimental ecologists can increasingly meet the causal assumptions required for valid causal inference. Importantly, DAGs allow researchers to transparently communicate their causal assumptions to others, which can facilitate more critical reception and lead to productive scientific debates that collectively deepen our understanding of ecological phenomena over time (e.g., Schoolmaster et al., 2020). Moreover, DAGs allow researchers to use their ecological domain knowledge, above all else, to build causal models, bridging the gap between ecological knowledge and statistical analysis. Ultimately, the uptake of this causal inference tool can significantly benefit experimental design, statistical analysis, and interpretation of results across experimental ecology.

## AUTHOR CONTRIBUTIONS

**Suchinta Arif:** Conceptualization (lead); investigation (equal); methodology (equal); project administration (equal); resources (equal); validation (equal); visualization (equal); writing – original draft (equal); writing – review and editing (equal). **Melanie Duc Bo Massey:** Conceptualization (supporting); formal analysis (equal); investigation (equal); methodology (equal); resources (equal); validation (equal); visualization (equal); writing – original draft (equal); writing – review and editing (equal).

## CONFLICT OF INTEREST STATEMENT

There is no conflict of interest to declare.

## Data Availability

Data sharing not applicable to this article as no datasets were generated or analyzed during the current study.

## References

[ece39947-bib-0001] Akaike, H. (1973). Information theory and an extension of the maximum likelihood principle. In B. N. Petrov & B. F. Csaki (Eds.), Second international symposium on information theory (pp. 267–281). Academiai Kiado.

[ece39947-bib-0002] Angilletta, M. J., Jr. , & Angilletta, M. J. (2009). Thermal adaptation: A theoretical and empirical synthesis. Oxford University Press.

[ece39947-bib-0003] Arif, S. , Graham, N. , Wilson, S. , & MacNeil, A. (2021). Causal drivers of climate‐mediated coral reef regime shifts. Ecosphere, 13(3), e3956.

[ece39947-bib-0004] Arif, S. , & MacNeil, A. (2022a). Predictive models aren't for causal inference. Ecology Letters, 25, 1741–1745. 35672133 10.1111/ele.14033

[ece39947-bib-0005] Arif, S. , & MacNeil, A. (2022b). Applying the structural causal model (SCM) framework for observational causal inference in ecology. Ecological Monographs, 93(1), e1554.

[ece39947-bib-0006] Cameron, S. F. , Wynn, M. L. , & Wilson, R. S. (2013). Sex‐specific trade‐offs and compensatory mechanisms: Bite force and sprint speed pose conflicting demands on the design of geckos (*Hemidactylus frenatus*). Journal of Experimental Biology, 216(20), 3781–3789. 23821718 10.1242/jeb.083063

[ece39947-bib-0007] Colinet, H. , Sinclair, B. J. , Vernon, P. , & Renault, D. (2015). Insects in fluctuating thermal environments. Annual Review of Entomology, 60, 123–140. 10.1146/annurev-ento-010814-021017 25341105

[ece39947-bib-0008] Cronin, J. , & Schoolmaster, D., Jr. (2018). A causal partition of trait correlations: Using graphical models to derive statistical models from theoretical language. Ecosphere, 9(9), e02422.

[ece39947-bib-0009] Donelson, J. M. , Salinas, S. , Munday, P. L. , & Shama, L. N. (2018). Transgenerational plasticity and climate change experiments: Where do we go from here? Global Change Biology, 24(1), 13–34. 29024256 10.1111/gcb.13903

[ece39947-bib-0010] Fuxjager, L. , Wanzenbock, S. , Ringler, E. , Wegner, K. , Ahnelt, H. , & Shama, L. (2019). Within‐generation and transgenerational plasticity of mate choice in oceanic stickleback under climate change. Philosophical Transactions of the Royal Society of London. Series B, Biological Sciences, 374(1768), 20180183. 30966960 10.1098/rstb.2018.0183 PMC6365864

[ece39947-bib-0011] Glymour, M. M. , & Greenland, S. (2008). Causal diagram. In K. J. Rothman , S. Greenland , & T. L. Lash (Eds.), Modern Epidemiology, 3rd ed. (pp. 183–209). Lippincott Williams & Wilkins.

[ece39947-bib-0012] Gottdenker, P. (1979). Francesco Redi and the fly experiments. Bulletin of the History of Medicine, 53(4), 575–592. 397843

[ece39947-bib-0013] Hariton, E. , & Locascio, J. (2018). Randomized controlled trials – The gold standard for effective research. BJOG, 125(13), 1716. 29916205 10.1111/1471-0528.15199 PMC6235704

[ece39947-bib-0014] Holland, P. (1986). Statistics and causal inference. Journal of the American Statistical Association, 81(396), 945–960.

[ece39947-bib-0015] Hunyadi, J. , Currier, T. , Modarres‐Sadeghi, Y. , Flammang, B. , & Clotfelter, E. (2020). Morphology, performance and fluid dynamics of the crayfish escape response. Journal of Experimental Biology, 223(15), jeb219873. 32561629 10.1242/jeb.219873

[ece39947-bib-0016] Huston, M. (1997). Hidden treatments in ecological experiments: Re‐evaluating the ecosystem function of biodiversity. Oecologia, 110, 449–460. 28307235 10.1007/s004420050180

[ece39947-bib-0017] Kimmel, K. , Dee, L. , Avolio, M. , & Ferraro, P. (2021). Causal assumptions and causal inference in ecological experiments. Trends in Ecology and Evolution, 36(12), 1141–1152. 34538502 10.1016/j.tree.2021.08.008

[ece39947-bib-0018] Kingsolver, J. G. , & Woods, H. A. (2016). Beyond thermal performance curves: Modeling time‐dependent effects of thermal stress on ectotherm growth rates. The American Naturalist, 187(3), 283–294. 10.1086/684786 26913942

[ece39947-bib-0019] Kinth, P. , Mahesh, G. , & Panwar, Y. (2013). Mapping of zebrafish research: A global outlook. Zebrafish, 10(4), 510–517. 24131434 10.1089/zeb.2012.0854 PMC3842892

[ece39947-bib-0046] Kobe, R. , Pacala, S. , & Silander, J. Jr (1995). Juvenile tree survivorship as a component of shade tolerance. Ecological Applications, 5(2), 517–532.

[ece39947-bib-0020] Korell, L. , Auge, H. , Chase, J. M. , Harpole, S. , & Knight, T. M. (2019). We need more realistic climate change experiments for understanding ecosystems of the future. Global Change Biology, 26, 325–327. 31412141 10.1111/gcb.14797

[ece39947-bib-0021] Koussoroplis, A. M. , Pincebourde, S. , & Wacker, A. (2017). Understanding and predicting physiological performance of organisms in fluctuating and multifactorial environments. Ecological Monographs, 87(2), 178–197.

[ece39947-bib-0022] Laubach, Z. , Murray, E. , Hoke, K. , Safran, R. , & Perng, W. (2021). A biologist's guide to model selection and causal inference. Proceedings of the Royal Society B: Biological Sciences, 288(1943), 20202815. 10.1098/rspb.2020.2815 PMC7893255 33499782

[ece39947-bib-0023] Lienart, G. , Mitchell, M. , Ferrari, M. , & McCormick, M. (2014). Temperature and food availability affect risk assessment in an ectotherm. Animal Behaviour, 89, 199–204.

[ece39947-bib-0024] Loreau, M. , Naeem, S. , Inchausti, P. , Bengtsson, J. , Grime, J. P. , Hector, A. , Hooper, D. U. , Huston, M. A. , Raffaelli, D. , Schmid, B. , Tilman, D. , & Wardle, D. A. (2001). Biodiversity and ecosystem functioning: Current knowledge and future challenges. Science, 294(5543), 804–808. 11679658 10.1126/science.1064088

[ece39947-bib-0025] Lusk, C. , & Del Pozo, A. (2002). Survival and growth of seedlings of 12 Chilean rainforest trees in two light environments: Gas exchange and biomass distribution correlates. Austral Ecology, 27, 173–182.

[ece39947-bib-0026] Massey, M. D. , & Hutchings, J. A. (2020). Thermal variability during ectotherm incubation: A review and synthesis. Journal of Experimental Zoology Part A, 335(1), 59–71. 10.1002/jez.2400 32767534

[ece39947-bib-0027] McElreath, R. (2020). Statistical rethinking: A Bayesian course with examples in R and Stan. CRC Press.

[ece39947-bib-0028] Mora, C. , Danovaro, R. , & Loreau, M. (2014). Alternative hypotheses to explain why biodiversity‐ecosystem functioning relationships are concave‐up in some natural ecosystems but concave‐down in manipulative experiments. Scientific Reports, 4, 5427. 24962477 10.1038/srep05427 PMC4069688

[ece39947-bib-0029] Morash, A. J. , Neufeld, C. , MacCormack, T. J. , & Currie, S. (2018). The importance of incorporating natural thermal variation when evaluating physiological performance in wild species. Journal of Experimental Biology, 221(14), jeb164673. 30037965 10.1242/jeb.164673

[ece39947-bib-0030] Morgan, R. , Finnøen, M. H. , Jensen, H. , Pélabon, C. , & Jutfelt, F. (2020). Low potential for evolutionary rescue from climate change in a tropical fish. Proceedings of the National Academy of Sciences, 117(52), 33365–33372. 10.1073/pnas.2011419117 PMC7776906 33318195

[ece39947-bib-0031] Pearl, J. (2009). Causality: Models, reasoning and inference (2nd ed.). Cambridge University Press.

[ece39947-bib-0032] Rollinson, N. , Holt, S. M. , Massey, M. D. , Holt, R. C. , Nancekivell, E. G. , & Brooks, R. J. (2018). A new method of estimating thermal performance of embryonic development rate yields accurate prediction of embryonic age in wild reptile nests. Journal of Thermal Biology, 74, 187–194. 29801626 10.1016/j.jtherbio.2018.03.008

[ece39947-bib-0033] Rubin, D. (1974). Estimating causal effects of treatments in randomized and nonrandomized studies. Journal of Educational Psychology, 66(5), 688–701.

[ece39947-bib-0034] Salinas, S. , Brown, S. C. , Mangel, M. , & Munch, S. B. (2013). Non‐genetic inheritance and changing environments. Non‐Genetic Inheritance, 1, 38–50.

[ece39947-bib-0035] Sato, K. , Shiomi, K. , Marshall, G. , Kooyman, G. , & Ponganis, P. (2011). Stroke rates and diving air volumes of emperor penguins: Implications for dive performance. Journal of Experimental Biology, 214(17), 2854–2863. 21832128 10.1242/jeb.055723

[ece39947-bib-0036] Schoolmaster, D. , Zirbel, C. , & Cronin, J. (2020). A graphical causal model for resolving species identity effects and biodiversity‐ecosystem function correlations. Ecology, 101(8), e03070. 32301506 10.1002/ecy.3070

[ece39947-bib-0037] Seebacher, F. , White, C. R. , & Franklin, C. E. (2015). Physiological plasticity increases resilience of ectothermic animals to climate change. Nature Climate Change, 5(1), 61–66.

[ece39947-bib-0038] Shadish, W. R. , Cook, T. D. , & Campbell, D. T. (2002). Experimental and quasi‐experimental designs for generalized causal inference. Mifflin and Company.

[ece39947-bib-0039] Shama, L. , & Wegner, K. (2014). Grandparental effects in marine sticklebacks: Transgenerational plasticity across multiple generations. Journal of Evolutionary Biology, 27(11), 2297–2307. 25264208 10.1111/jeb.12490

[ece39947-bib-0040] Spinks, R. , Bonzi, L. , Ravasi, T. , Munday, P. , & Donelson, J. (2020). Sex‐ and time‐specific parental effects of warming on reproduction and offspring quality in a coral reef fish. Evolutionary Application, 14(4), 1145–1158. 10.1111/eva.13187 PMC8061261 33897826

[ece39947-bib-0041] Taylor, E. N. , Diele‐Viegas, L. M. , Gangloff, E. J. , Hall, J. M. , Halpern, B. , Massey, M. D. , Rödder, D. , Rollinson, N. , Spears, S. , Sun, B. J. , & Telemeco, R. S. (2021). The thermal ecology and physiology of reptiles and amphibians: A user's guide. Journal of Experimental Zoology Part A: Ecological and Integrative Physiology, 335(1), 13–44. 32638552 10.1002/jez.2396

[ece39947-bib-0042] Tilman, D. , Wedin, D. , & Knops, J. (1996). Productivity and sustainability influenced by biodiversity in grassland ecosystems. Nature, 379(6567), 718–720.

[ece39947-bib-0043] Wieser, W. (1973). Effects of temperature on ectothermic organisms. Springer.

[ece39947-bib-0044] Williams, T. , Bach, C. , Matthiesen, N. , Henriksen, T. , & Gagliardi, L. (2018). Directed acyclic graphs: A tool for causal studies in paediatrics. Pediatric Research, 84, 487–493. 29967527 10.1038/s41390-018-0071-3 PMC6215481

[ece39947-bib-0045] Zizzari, Z. V. , & Ellers, J. (2014). Rapid shift in thermal resistance between generations through maternal heat exposure. OIKOS, 123(11), 1365–1370.

